# Cohorts in chronic disease research: experiences from the PRO-LIFE cohort, Varkala, South India

**DOI:** 10.1186/1753-6561-7-S5-O7

**Published:** 2013-08-30

**Authors:** V Raman Kutty

**Affiliations:** 1 Sree Chitra Tirunal Institute for Medical Science and Technology, Trivandrum, India

## 

This study demonstrates the setting up of a cohort study in a resource poor setting. The study began as an initial survey in 2001 conducted at Varkala, Kerala by Health Action by People, a non-profit research group. It was a cross sectional study of all households in seven *Panchayaths* and an urban segment coming under the ICDS (Integrated Child Development Scheme) block.

Eventually with multiple funding sources, the study was extended as a cohort study, in the form of a registry for births, deaths and major health events in the community from 2002- 2006. The cohort was named PRO-LIFE (Population registry of lifestyle diseases) with the objectives of setting up a community registry for coronary disease, hypertension, stroke, COPD & type 2 diabetes and to initiate community level action for prevention of these diseases.

The study was conducted by trained grass root level workers of the ICDS programme and the comprehensive survey included socio-demographic characteristics, food frequency information, detailed lifestyle attributes and information on births and deaths. A cause of death analysis was also conducted during the period 2002-2006 which showed a similarity with USA except neoplasms. However the age standardised death rates were much higher than USA. Other findings include a high suicide rate among the young population, higher prevalence of diabetes in the affluent group and a reduced relative risk for mortality from coronary heart disease in more physically active men and women.

The follow up was stopped after 2006 due to lack of funds and unreliable collection of information. It is currently proposed to resurvey the project population including anthropometry and biochemical profile with periodic follow up and fool proof system of reporting births, deaths and major events. The experience showed that full support of the local self government and political units is very important. Existing health workers need to be carefully selected for long term follow up. Discussion points included the reasons for high suicide rate among young females and the social gradient of non-communicable diseases. The high rate of suicide among youngsters may be due to their high aspirations and pressures such as entrance examinations. The higher prevalence of non-communicable diseases in the affluent classes may be due to their lifestyle, however it may change very soon as we move from a situation of food scarcity to plenty of food.

